# The *I-MICRO trial*, Ilomedin for treatment of septic shock with persistent microperfusion defects: a double-blind, randomized controlled trial—study protocol for a randomized controlled trial

**DOI:** 10.1186/s13063-020-04549-y

**Published:** 2020-07-01

**Authors:** Matthieu Legrand, Hafid Ait Oufella, Daniel De Backer, Jacques Duranteau, Marc Leone, Bruno Levy, Patrick Rossignol, Eric Vicaut, François Dépret, for the I-MICRO trial investigators, François Depret, Jean-Michel Constantin, Hafid Ait Oufella, Daniel De Backer, Bruno Levy, Marc Leone, Jacques Dureanteau, Samuel Gaugain, Jules Audart, Jean-Yves Lefrant, Bruno Megarbane, Julien Pottecher, Romain Sonneville, Thomas Rimmele, Carole Ichai, Antoine Vieillard, Alexy Tran Dinh, Cécile Aubron, Arnaud Mari, Vincent Labbe, Gaetan Plantefeve, Anne Laure Fedou, Damien Barraud, Stéphane Gaudry, Helene Nougue

**Affiliations:** 1 Department of Anaesthesiology, Critical Care Medicine and Burn Unit, AP-HP, Saint Louis and Lariboisière University Hospitals, 2 rue A. Paré, 75010 Paris, France; 2 INSERM UMR-S942, Institut National de la Santé et de la Recherche Médicale (INSERM), Lariboisière Hospital and INI-CRCT Network, Paris, France; 3 Univ Paris Diderot, F-75475 Paris, France; 4 Department of Anesthesia and Perioperative Care, University of California, San Francisco, 500 Parnassus Avenue MUE416, Box 0648, San Francisco, CA 94143 USA; 5 Assistance Publique - Hôpitaux de Paris (AP-HP), Hôpital Saint-Antoine, Service de Réanimation Médicale, 75571 Paris Cedex 12, France; 6 Sorbonne Université, Université Pierre-et-Marie Curie, Paris 6, France; 7 Intensive Care Department, CHIREC Hospitals, Université Libre de Bruxelles, Brussels, Belgium; 8 Department of Anesthesia and Intensive Care, Hôpitaux Universitaires Paris Sud, Université Paris Sud XI, Le Kremlin Bicêtre, France; 9 Aix Marseille Université, Assistance Publique Hôpitaux de Marseille, Service d’Anesthésie et de Réanimation, Hôpital Nord, Marseille, France; 10 Service de Réanimation Médicale, Centre Hospitalo-Universitaire de Nancy, F-54511 Vandœuvre-Lès-Nancy, France; 11 Université de Lorraine, F-54000 Nancy, France; 12 Centre d’Investigation Clinique Plurithématique Pierre Drouin-INSERM CHU de Nancy, Nancy, France; 13 FCRIN INI-CRCT (Cardiovascular and Renal Clinical Trialists) Network, Université de Lorraine, Nancy, France; 14 APHP, Department of Biostatistics, Université Paris-Diderot, Sorbonne-Paris Cité, Fernand Widal Hospital, Paris, France

**Keywords:** Sepsis, Iloprost, Prostacyclin, Vasodilator, Microcirculation, Skin mottling, Capillary refill time, Outcome

## Abstract

**Background:**

Septic shock remains a significant cause of death in critically ill patients. During septic shock, some patients will retain microcirculatory disorders despite optimal hemodynamic support (i.e., fluid resuscitation, vasopressors, inotropes). Alterations in the microcirculation are a key pathophysiological factor of organ dysfunction and death in septic shock patients. Ilomedin is a prostacyclin analog with vasodilatory effect and anti-thrombotic properties (i.e., inhibition of platelet aggregation) preferentially at the microcirculatory level. We hypothesize that early utilization of intravenous Ilomedin in septic shock patients with clinical persistence of microperfusion disorders would improve the recovery of organ dysfunction.

**Methods:**

The I-MICRO trial is a multicenter, prospective, randomized, double-blinded, placebo-controlled study. We plan to recruit 236 adult patients with septic shock and persistent microcirculatory disorders (i.e., skin mottling or increased capillary refill time) despite hemodynamic support. Participants will be randomized to receive a 48-h intravenous infusion of either Ilomedin or placebo starting at the earliest 6 h and later 24 h after septic shock. The primary outcome will be the change (delta) of sequential organ failure assessment (SOFA) score between randomization and day 7. Secondary outcomes will include mean SOFA score during the first 7 days after randomization, mortality at day 28 post-randomization, number of ventilation-free survival days in the 28 days post-randomization, number of renal replacement therapy-free survival days in the 28 days post-randomization, number of vasopressor-free survival days in the 28 days post-randomization, and mottling score at day 1 after randomization.

**Discussion:**

The trial aims to provide evidence on the efficacy and safety of Ilomedin in patients with septic shock and persistent microcirculatory disorders.

**Trial registration:**

NCT NCT03788837. Registered on 28 December 2018

## Background

Septic shock remains a significant cause of death in critically ill patients [1]. Controlling the source of infection, appropriate and early antibiotics administration, and supportive care are the key elements of septic shock treatment [2]. Alterations in microcirculation are a critical pathophysiological factor of organ dysfunction and death in septic shock patients [3, 4]. Several clinical studies have revealed that microcirculatory disorders are associated with poor outcome in septic shock [5–8]. During septic shock, some patients will retain microcirculatory disorders despite optimal hemodynamic support (fluid resuscitation, vasopressors, inotropes). Preclinical data have furthermore strongly suggested that recruiting the microcirculation could improve organ perfusion and function in sepsis [4]. However, to date, no treatment specifically targeting the microcirculation has been shown to improve the patient’s outcome. Reasons for the microcirculatory defects are the release of local vasoconstrictive agents and intravascular microthrombi. Persistence of mottling, prolonged skin recoloration time, and cyanosis of the extremities are the easily and frequently observed manifestations of these microcirculatory disorders [9]. Ilomedin is a prostacyclin analog with a potent vasodilatory effect together with anti-thrombotic properties (inhibition of platelet aggregation) [10]. Improvement in mesenteric perfusion has, moreover, been observed in experimental sepsis using Ilomedin [11, 12]. Our group has reported that administration of Ilomedin in patients with refractory septic shock was associated with a rapid and sustained improvement in peripheral perfusion [13]. Altogether, Ilomedin may prevent organ dysfunction or improve its recovery in septic shock patients and ultimately improve outcomes. This study aims to assess the effect of Ilomedin infusion on organ function in septic shock patients with persistent peripheral hypoperfusion despite standard of care resuscitation.

## Methods/design

### Aim, design, and setting of the study

**Fig. 1:**
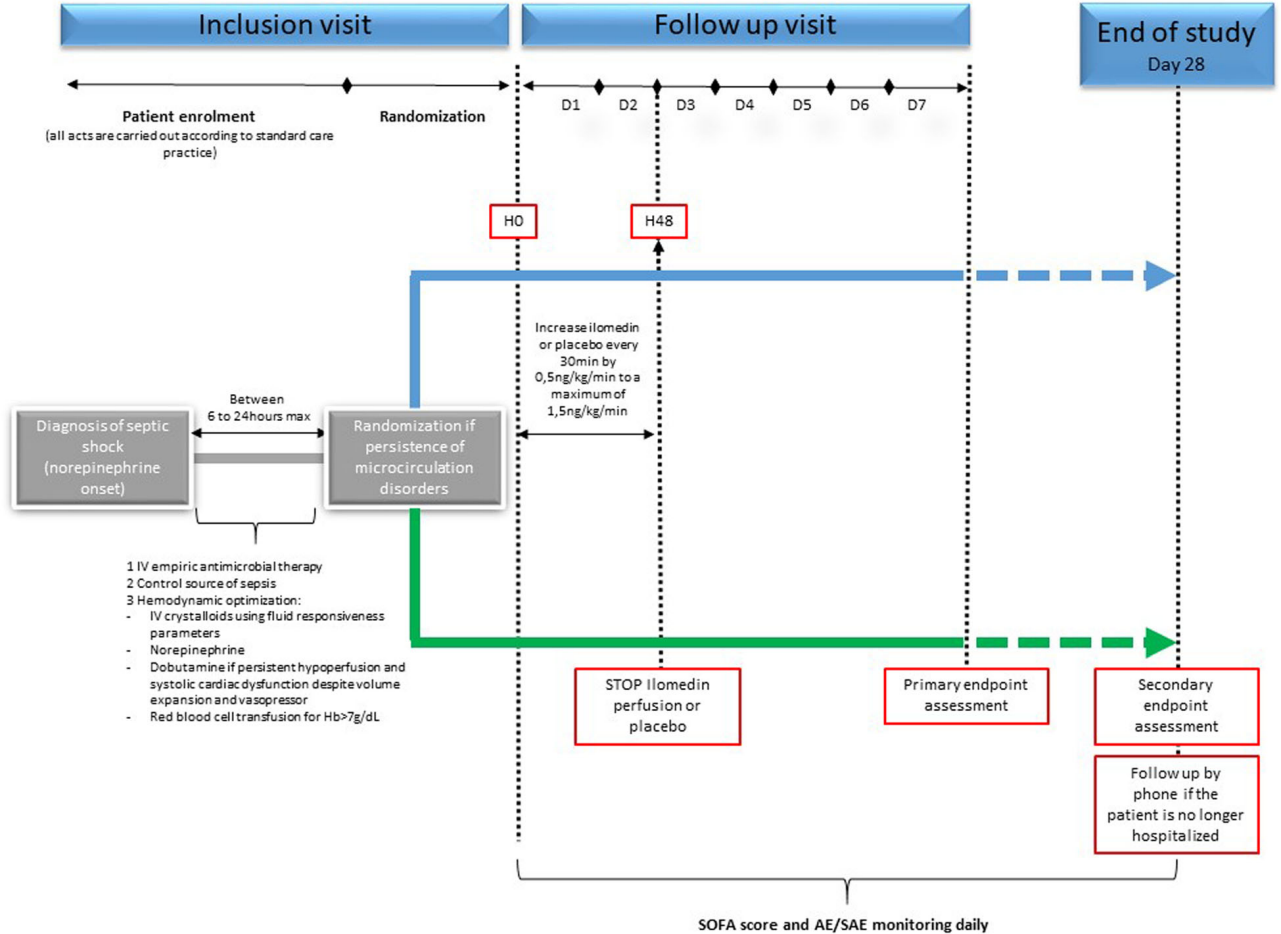
Study design. SOFA score, sequential organ failure assessment score; AE, adverse events; SAE, serious adverse events

The study protocol is in accordance with the SPIRIT guidelines [14] (Fig. 1). I-MICRO is an academic, investigator-initiated multicenter (~ 25 centers), prospective, parallel-group (two groups), double-blinded, placebo-controlled, randomized trial in adults with septic shock admitted to an intensive care unit. This trial’s aim is whether the administration of Ilomedin could improve organ function compared to placebo. Exploratory secondary outcomes and adjusted analyses. A total of 236 evaluable patients with septic shock and persistence of microcirculation disorders (i.e., skin mottling or increased capillary refill time) will be enrolled in 25 centers with experience in the management of patients with septic shock (list of participating centers is in Appendix 1).

The primary outcome is the difference between SOFA scores from randomization to day 7 in patients allocated to Ilomedin and in patients allocated to placebo.

The secondary outcomes are as follows:

- Mortality at day 28
- Mean SOFA score during the first 7 days after randomization
- Number of survival days in the 28 days post-randomization
- Number of ventilation-free survival days in the 28 days post-randomization
- Number of renal replacement therapy-free survival days in the 28 days post-randomization
- Number of vasopressor-free survival days in the 28 days post-randomization
- Mottling score at day 1 after randomization

### Characteristics of participants

All participants will be included and randomized by the clinician in charge of the patient. To be included, patients must meet all the following inclusion criteria:

1. Patients over 18 years of age with signed informed consent or inclusion under the emergency provisions of the law (Article L1122-1-3 of the PHC/modified by order no. 2016-800 of June 16, 2016 - art. 2)
2. Patients in septic shock defined by the third international definition [15]:
  ◦ Suspected or proven infection
  ◦ Organ dysfunction defined by an acute change in total SOFA score ≥ 2
  ◦ And serum lactate level > 2 mmol/L despite standard of care hemodynamic optimization
  ◦ Persistent hypotension requiring vasopressor treatment despite standard of care hemodynamic optimization and mean arterial pressure maintained > 65 mmHg
3. Persistence of peripheral hypoperfusion (skin mottling and/or finger skin recoloration time > 3 s and/or knee skin recoloration time > 4 s) within 6 to 24 h after norepinephrine onset

Patients will be excluded if any of the following criteria apply:

1. Refusal to participate in the study
2. Pregnancy and breastfeeding
3. Hypersensitivity to Ilomedin or any of the excipients
4. Conditions where the hemorrhagic risk may be increased due to the effects of Ilomedin on platelets (i.e., evolving hemorrhage, trauma, intracranial hemorrhage, active gastric ulcer)
5. Platelet count < 30,000/mm3
6. Unstable angina
7. Severe cardiac rhythm disorders since norepinephrine onset (ventricular fibrillation or tachycardia)
8. Severe hypoxemia (PaO_2_/FiO_2_ < 100)
9. Myocardial infarction in the last 6 months
10. Lack of social insurance
11. Persons deprived of liberty

The patients screened for randomization will present septic shock according to the new Sepsis-3 definition and signs of peripheral hypoperfusion (mottling score ≥ 2 and/or finger skin recoloration time > 3 s, and/or knee skin recoloration time > 4 s) as a surrogate parameter for microcirculatory hypoperfusion. A video has been shown to all investigators (Supplementary files 1 and 2). The study centers will treat patients according to the standard of care [2]. However, the following checklist for hemodynamic optimization concerning the standard of care before randomization:

1. An initial fluid challenge should achieve a minimum of 30 mL/kg of crystalloids (a portion of this may be albumin equivalent).
2. The fluid challenge technique will be applied wherein fluid administration is continued as long as there is a hemodynamic improvement based on dynamic (e.g., change in pulse pressure, stroke volume variation) or static (e.g., arterial pressure, heart rate) variables.
3. Norepinephrine is titrated to target a mean arterial pressure of 65 mmHg in patients without a history of chronic hypertension and 75 mmHg in patients with a history of hypertension if the patient is not currently receiving renal replacement therapy.
4. Dobutamine will be initiated if cardiac dysfunction is diagnosed (i.e., left ventricular ejection fraction < 40% on echocardiography and/or low cardiac index < 2.5 L/min/m^2^, increased filling pressure-wedge pressure > 18 mmHg on pulmonary artery catheter, and/or ScvO_2_ < 70% or SvO_2_ < 60% in a patient unresponsive to fluid challenge).
5. Transfusion threshold will be a hemoglobin target of 7–8 g/dL

### Randomization process

Patients in the trial treatment are allocated to treatment with Ilomedin or placebo in a ratio of 1:1. The randomization list will be performed at the CRU “Lariboisière-St Louis,” block-free, and stratified by center. The randomization list will be developed by a different biostatistician than the biostatistician who will conduct the final analysis within the CRU “Lariboisière- St Louis.”

Randomization is carried out by assigning treatment boxes in ascending order of the numbers available on site. Each center receives four treatment boxes with a randomization number on each box. Two new boxes will be sent to centers when they have recruited two participants. To randomize a patient and allocate treatment, the investigator takes the box with the smallest randomization number.

### Ilomedin

The intervention is the intravenous administration of Ilomedin or to placebo (0.9% saline) via a central venous catheter. The perfusion will start at the first dose of 0.5 ng/kg/min with increments every 30 min up to a maximum of 1.5 ng/kg/min. The study drug or placebo is administered over 48 h, at the earliest 6 h after the diagnostic of septic shock (i.e., norepinephrine onset) and at last 24 h after the diagnostic.

Ilomedin is supplied in 50 μg/0.5 mL vials. A trained unblinded person reconstitutes the syringe with 50 mL of 0.9% saline to provide a ready-to-use solution. The final concentration of the drug substance Ilomedin is 1 μg/mL. Eight syringes are used for each patient (syringes are changed every 6 h, even if not empty).

The doses for each patient are calculated automatically according to the individual’s ideal body weight, using information from the electronic case report form (eCRF). The calculation results in an individual infusion rate for each level (i.e., 0.5, 1, and 1.5 ng/kg/min) for each patient. Patients, investigation site staff, site research coordinators, persons performing the assessments, the sponsor, central mottling pictures assessor, the staff in charge of treating the patients, and data managers will be blinded to the treatment allocation except for the independent, unblinded statistician approving the randomization scheme. The identification of treatment will be concealed by using a matching placebo to the study product that will be identical in packaging, labeling, and appearance. Unblinding will be requested for any reason considered essential by the investigating physician.

In systemic mean arterial pressure decreases below 65 mmHg after initiation of the treatment, we propose the following algorithm:

1. The need for fluid loading will be assessed using dynamic parameters (i.e., pulse pressure or stroke volume variations when applicable, end-expiratory occlusion test) or fluid challenge.
2. An increase of 25% in norepinephrine of baseline dose will be allowed. If systemic arterial hypotension persists, the infusion rate will be decreased to the previous dose (e.g., 1.5 to 1 ng/kg/min or 0.5 ng/kg/min to zero) every 15 min

In the case of profound hypotension (mean arterial pressure below 50 mmHg) for more than 5 min without identified etiology (e.g., sedation bolus), the treatment will be interrupted.

If hypoxemia occurs after introducing the treatment (or placebo), FiO_2_ can be increased to reach a SpO_2_ > 88–92%; if profound hypoxemia occurs (PaO_2_/FiO_2_ < 100) after increasing the infusion rate, the treatment will be decreased to the previous dose (e.g., 1.5 to 1 ng/kg/min or 0.5 ng/kg/min to zero) every 15 min.

In case of severe bleeding after randomization (active hemorrhage with hemodynamic instability), the treatment will be discontinued and not resumed.

If the patient requires emergency surgery, the treatment will be interrupted before transfer to the operating room. The treatment can be resumed 6 h after the end of surgery if peripheral hypoperfusion persists for a total duration of treatment (including the preoperative phase) of 48 h.

The administration period is followed by a clinical follow-up period of 28 days after randomization (Fig. 1).

No treatment will be prohibited concerning the research. However, due to the platelet anti-aggregate effect of Ilomedin, some concomitant treatments must be associated with precaution, especially anti-coagulants and platelet anti-aggregate treatments.

### Data collection

Data on all patients will be collected by trained study nurses or physicians using a web-based e-CRF and stored in a secured server (Telemedicine Technologies, Boulogne-Billancourt, France). Data collected and time points are presented Table 1. Monitoring is performed by the clinical research organization and the sponsor.

**Table 1:** SPIRIT schematic schedule of enrolment, interventions, and assessments

	Inclusion and randomization visit	Follow up visits, day 1 to day 7	End of study, day 28
Inclusion and non-inclusion criteria	X		
Informed consent	X	X^(0)^	X^(0)^
Randomization	X		
Medical history/comorbidities	X	X	X*
Concomitant treatment	X	X	X*
Clinical examination	X^(1)^	X^(1)^	X* ^(1)^
Blood sample for local biological assessment	X^(2)^	X^(2)^	
Glasgow Coma Score	X	X	X*
Assessment of SOFA score	X	X	
Molting score (picture of skin knees)	X	X**	
Capillary refill time	X	X	
biological collection	X, within the 12 first hours after randomization		
Drug intake	X^(3)^	X^(3)^	
Retrieval of adverse events	X	X	X
Assessment of morbidity and mortality	X	X	X

Intravenous administration of Ilomedin or placebo will be started at 0.5 ng/kg/min increasing every 30 min up to a maximum of 1.5 ng/kg/min for 48 h

^(0)^ If not done at the previous visits (according to law L1122-1-3 of the PHC)

^(1)^ Clinical examination:

- Hemodynamic parameters: systolic, mean, and diastolic arterial pressure; heart rate; central venous pressure; central venous oxygen saturation; cardiac output if available

- Electrocardiogram

^(2)^ Blood sample:

- Biological parameters: arterial plasma lactate level, plasma pH and base excess, PaO_2_ and PaO_2_/FiO_2_, PaCO_2_, blood urea nitrogen, serum creatinine, serum potassium level, hemoglobin, brain natriuretic peptide (BNP) or NT-ProBNP, ultrasensitive troponin, total bilirubin level and platelet count

*if the patient is still hospitalized

**at inclusion and at D2

^(3)^ Drug intake: the treatment period is 48 h

All information required by the protocol will be provided in the case report form and an accompanying explanation given by the investigator for each missing data.

The data will be transferred in the case report forms as and when they are obtained, whether clinical, labs, or imaging. Erroneous data tracked on case report forms will be replaced by a declared investigator, which ensures the confidentiality of the data and authenticates the interventions.

Daily assessments (e.g., SOFA score) until day 7 and secondary outcomes will be performed by the respective center investigators. All data and other information generated will be held in strict confidence. The patients will be identifiable only by their initials and number. The Standard Protocol Items: Recommendation for Interventional Trials (SPIRIT) reporting guidelines are applied [14] (SPIRIT checklist is provided as supplementary File 3). Data from the trial will be made available to the reviewers upon request by the editor of the journal.

For ancillary studies, blood will be collected (15 mL) at the same time as the sample routinely collected within the 12 first hours after randomization. The aliquots previously labeled and stowed in the specific boxes for the study will be stored at − 80 °C, under the responsibility of the principal investigator of each center. Then, every 6 months, the aliquots will be shipped to the “Centre de Ressources Biologiques” (CRB) of the Lariboisière Hospital.

### Statistical analysis

This trial’s primary aim is to demonstrate superiority in the intent-to-treat analysis of Ilomedin versus placebo on delta SOFA_J0–J7_. The null hypothesis is that there are no differences in delta SOFA_J0–J7_ between the two treatment groups.

Delta SOFA_J0–J7_ will be compared between the two groups using the non-parametric Mann-Whitney test. The efficacy of Ilomedin will be considered if the null hypothesis for the primary endpoint is rejected and if the treatment difference is in favor of Ilomedin in the sense of a shift to higher delta SOFA_J0–J7_.

We need to include *N* = 117 patients per group to have an 80% power to detect a difference between the two groups corresponding to a 1 point difference in delta SOFA between the two groups. Continuous variables will be summarized using the number of observations; mean; standard deviation; minimum; maximum; 25%, 50%, and 75% quartiles; and the two-sided 95% confidence intervals. Means, medians, minimum, maximum, and standard deviations will be presented to one further decimal place.

Categorical variables will be expressed as absolute and relative frequencies (percentages). Percentages will be rounded to one decimal place, and there may be occasions where the total of the percentages does not exactly equal 100%. Missing values will be imputed by multiple imputation techniques.

### Data monitoring and interim analysis

A steering committee provides scientific direction for the trial and meets periodically to assess its operational progress every 9 months. It also provides scientific input and addresses policy issues regarding the protocol. The committee chair is responsible for communicating with the Data Safety and Monitoring Committee (DSMC) when appropriate. The Steering Committee is comprised of the coordinating investigator, Dr. François Dépret; scientific officer, Pr Matthieu Legrand; the statistician, Pr Eric Vicaut; a representative of the sponsor (AP-HP DRCI), Elodie Lemadre; and members selected on the basis of their outstanding expertise in the field and contribution to the study, Pr Daniel De Backer, Pr Bruno Levy, Pr Marc Leone, Pr Jacques Duranteau, and Pr Hafid Ait Oufella.

An independent DSMC monitors the quality of the trial and has access to the trial outcome and accumulated safety data, including serious adverse events (SAEs), suspected unexpected severe adverse reactions, and mortality. The composition of the DSMC is presented in Appendix 2. Besides, the DSMC will review the safety data from a clinical and safety point of view on an on-going basis.

An interim safety analysis will be scheduled once the first 50 patients have been included and have completed their 28-day clinical observation phase. The data analyst for the primary analyses will be blinded. An interim analysis will be made after evaluating 50% of patients by an unblinded statistician and reviewed by the DSMC based on clean data on the primary and secondary target variables and the latest status of safety data. This analysis will allow three possible recommendations of the DSMC to the sponsor:

- Early stop of the study if the *p* value for the main criterion is less than *p* = 0.0035 (according to O’Brien-Fleming boundaries)
- Sample size reassessment (only increase in sample size should be accepted (calculated from the ADDPLAN software))
- Stopping for futility if the predictive power is less than 50%

The nominal alpha value for the interim analysis of the main criterion will be equal to 0.0035 and to 0.049 for the final analysis of this criterion (according to O’Brien-Fleming boundaries). All other tests will be two-sided at a 5% significance level.

The investigator will assess the severity of each adverse event and record all serious and non-serious adverse events in the case report form (Appendix 3). The investigator will also assess the potential causal relationship between the serious adverse events and the drug. Serious adverse events require a notification without delay by the investigator to the sponsor.

The sponsor will notify all the investigators any information that could adversely affect the safety of the participants. Assistance Publique Hôpitaux de Paris (AP-HP) is the sponsor of this study and has delegated power to its Clinical Research and Development Department (DRCD) to conduct the study under Article L.1121-1 of the French Public Health Code. AP-HP reserves the right to terminate the study at any time for medical or administrative reasons. In this case, the investigator will be informed accordingly.

**Fig. 2:**
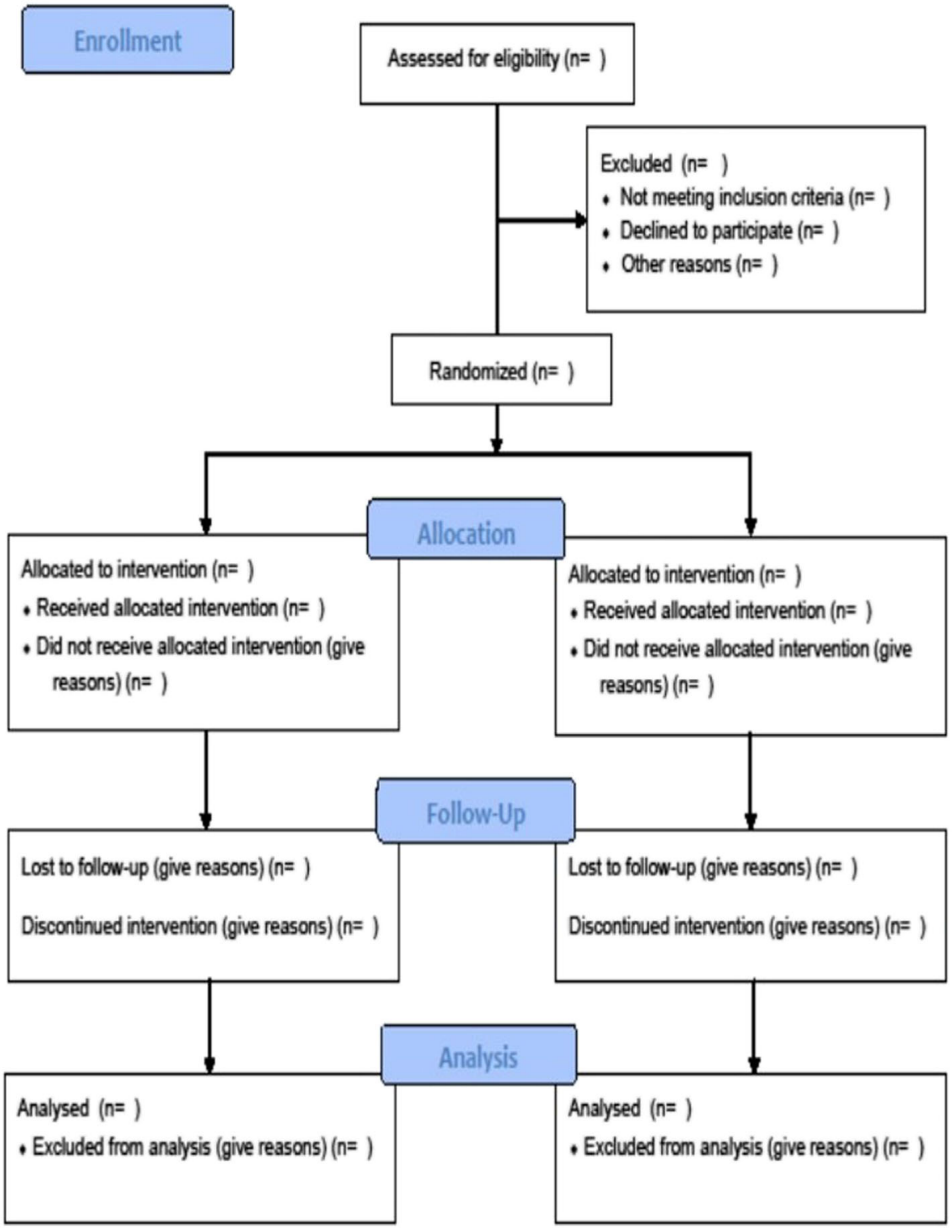
CONSORT flow chart of the study

In the case of lost to follow-up, the investigator will do his or her best to contact him or her in order to know whether he is alive. Mailing address, phone number, and phone number of at least one relative will be collected at inclusion to contact the patient. The contact details of the patient and the trusted person are recorded in the patient’s medical record and the eCRF. Furthermore, all patients are recorded in the French social security system and can be followed. If an individual leaves the research prematurely, participant data can be used unless an objection was recorded when the patient signed the consent form. If the consent was withdrawn, no data about the individual could be used unless stated otherwise. In practice, the participant is excluded from the research (Fig. 2).

It should be noted that all amendments will be validated by the institutional review board. Once validated, the content of the amendments will be communicated to all investigators through contact emails, newsletters (on a three-month basis), and investigators’ meeting.

In accordance with good clinical practices, the sponsor is responsible for obtaining the permission of all parties involved in the research to guarantee direct access to all locations where the research will be carried out, to the source data, and to the source documents and the reports, with the goal of quality control and audit by the sponsor. Furthermore, the investigators will make available to those in charge of data monitoring, quality control, and audit relating to the biomedical research, the documents, and personal data strictly necessary for these controls, under the legislative and regulatory provisions in force (Articles L.1121-3 and R.5121-13 of the French Public Health Code).

## Discussion

Septic shock with persistent peripheral perfusion disorders is associated with a very poor prognosis with no specific pharmaceutical therapies. The administration of Ilomedin has the potential to improve clinical outcomes through the prevention of organ failure. The I-MICRO trial aims to detect a beneficial effect of Ilomedin on organ function in patients with septic shock and persistent peripheral perfusion disorders or to provide the basis for an additional pivotal trial. Other important clinical outcomes, including survival will be explored as secondary endpoints. The design of this study lies in the identification of patients who are most likely to benefit from adjunctive treatment recruiting the microcirculation, i.e., remaining with altered perfusion despite standard of care. Mottling and increase capillary refilling time have previously been associated with alteration of the microcirculation in septic shock. Their observation is furthermore associated with poor outcomes. The ease of measuring these clinical symptoms without additional test or device makes them generalizable criteria to decide rescue strategies in sepsis. The results of this trial should provide essential and critical information on the feasibility, safety, and efficacy for preventing organ failure or for recovery from organ failure in this sub-population of septic shock with a very high risk of death. This trial will also provide essential data for designing a future larger clinical trial with mortality as the primary endpoint in this population.

### Trial status

The current protocol is version 3.0, dated December 25, 2019. Recruitment began on July 3, 2019. The approximate completion date for recruitment is in July 2022. This trial was prospectively registered before the recruitment began. The study protocol was approved by the institutional review board (IRB) of Sud-Est V on October 30, 2018 (approval number 2018-001709-10) and from the Agence Nationale de Sécurité du Medicament et des Produits de santé (MEDAECNAT-2018-07-00015) and was registered in the clinical trial on December 28, 2018 (NCT03788837) and in EudraCT (N° 2018-001709-10) (Supplementary files 4 and 5). The Scientific director will be the first author, the principal investigator last author, and the methodologist the penultimate one. Each site principal investigator will be listed as an author from the second author based on the number of patients included on each site (if at least one patient is included from the site). All other investigators will be listed as collaborators.

### Supplementary information

**Additional file 1:.** Supplementary video 1.

**Additional file 2:.** Supplementary video 2.

**Additional file 3:.** SPIRIT 2013 Checklist: Recommended items to address in a clinical trial protocol and related documents.

**Additional file 4:.** Clinical trial authorization for medicinal products for human use

**Additional file 5:.** Protection of persons committee.

## Data Availability

Data from the trial will be made available to the reviewers upon request by the editor of the journal.

## Appendix 1

**Table 2:** I-MICRO trial principal and deputy investigators

Nom	Prénom	Ville	CHU	Service	Mail	Tel
**DEPRET**	**François**	**Paris**	**Saint louis**	**Surgical ICU**	francois.depret@aphp.fr	01 42 49 95 70
CONSTANTIN	Jean-Michel	Paris	La Pitié Salpetrière	Surgical ICU	jean-michel.constantin@aphp.fr	01 72 16 56 41
AIT OUFELLA	Hafid	Paris	CHU St-Antoine	ICU	hafid.ait-oufella@aphp.fr	01 49 28 23 62
DE BACKER	Daniel	Waterloo (be)	Hôpital de Braine	Mixed ICU	ddebacke@ulb.ac.be	(+)32 2 434 93 24
LEVY	Bruno	Nancy	CHU Nancy-Brabois	Medcial ICU	blevy5463@gmail.com	03 83 75 44 69
LEONE	Marc	Marseilles	CHU Hôpital Nord	ICU	marc.leone@ap-hm.fr	04 91 96 86 50
DUREANTEAU	Jacques	Paris	CHU Kremlin-Bicêtre	Surgical ICU	jacques.duranteau@aphp.fr	01 45 21 24 19
GAUGAIN	Samuel	Paris	CHU St-Louis/ Lariboisière	Surgical ICU	samuel.gaugain@aphp.fr	01 49 95 80 85
AUDART	Jules	Clermont-Ferrand	CHU Clermont-Ferrand	Surgical ICU	jaudart@chu-clermontferrand.fr	04 73 75 05 01
LEFRANT	Jean-Yves	Nîmes	CHU Carémeau	Surgical ICU	jean.yves.lefrant@chu-nimes.fr	04 66 68 30 50
MEGARBANE	Bruno	Paris	CHU Lariboisière	ICU	bruno.megarbane@lrb.aphp.fr	01 49 95 84 42
POTTECHER	Julien	Strasbourg	CHU Hautepierre	ICU	julien.pottecher@chru-strasbourg.fr	03 88 12 70 95
SONNEVILLE	Romain	Paris	CHU Bichat	Medcial ICU	romain.sonneville@aphp.fr	01 40 25 61 39
RIMMELE	Thomas	Lyon	Edouard Herriot	Surgical ICU	thomas.rimmele@chu-lyon.fr	04 72 11 69 88
ICHAI	Carole	Nice	CHU Nice	Mixed ICU	ichai@unice.fr	04 92 03 36 27
VIEILLARD	Antoine	Boulogne-Billancourt	CHU Ambroise Paré	ICU	antoine.vieillard-baron@aphp.fr	01 49 09 55 77
TRAN DINH	Alexy	Paris	CHU Bichat	Surgical ICU	alexy.trandinh@gmail.com	01 40 25 83 55
AUBRON	Cécile	Brest	CHU Brest	Medcial ICU	cecile.aubron@chu-brest.fr	02 98 34 71 81
MARI	Arnaud	Saint-Brieuc	CH Yves Le Foll	Mixed ICU	monsieurarnaudmari@gmail.com	02 90 01 70 60
LABBE	Vincent	Paris	CHU Tenon	Mixed ICU	vincent.labbe@aphp.fr	01 56 01 65 72
PLANTEFEVE	Gaetan	Argenteuil	CH Victor Dupouy	Mixed ICU	gaetan.plantefeve@ch-argenteuil.fr	01 34 23 24 51
Fedou	Anne Laure	Limoges	C H U DUPUYTREN	ICU	Anne-laure.fedou@chu-limoges.fr	05 55 05 58 41
Barraud	Damien	Metz	Hopital de Mercy - CHR Metz Thionville	Mixed ICU	d.barraud@chr-metz-thionville.fr	03.87.18.62.34
GAUDRY	Stéphane	Bobigny	CHU Avicenne	Mixed ICU	Stephane.gaudry@aphp.fr	01 48 95 52 41
Nougue	Helene	Paris	HEGP	Surgical ICU	Helene.nougue@aphp.fr	01 56 09 25 20

## Appendix 2

### I-MICRO trial Data and Safety Monitoring Committee

Pr Charle Edouard Luyt, Medical Intensivist, Paris, France

Pr Etienne Gayat, Statistician, Paris, France

Pr Atul Pathak, Pharmacologist, Toulouse, France

Pr Olivier Sitbon, Pneumologist, Le Kremlin-Bicêtre, France

## Appendix 3

### List of events and serious adverse events

The investigator must document the serious adverse event as thoroughly as possible and provide the medical diagnosis, if possible. The investigator assesses the severity of the adverse events by using more general terms:

- *Mild: tolerated by the patient, does not interfere with daily activities*
- *Moderate: sufficiently uncomfortable to affect daily activities*
- *Serious: preventing daily activities*

The investigator must assess the *causal relationship* between the serious adverse events and the investigational medicinal product(s).

The method used by the investigator is based on the WHO Uppsala Monitoring Center method and uses the following causality terms:

- Certain
- Probable/likely
- Possible
- Unlikely (not ruled out)

These terms are defined as follows (extracted from the WHO-UMC causality categories, version dated 17 April 2012).

**Table 3:** WHO-UMC causality categories

Causality term	Assessment criteria
Certain	• Event or laboratory test abnormality, with plausible time relationship to drug intake • Cannot be explained by disease or other drugs • Response to withdrawal plausible (pharmacologically, pathologically) • Event definitive pharmacologically or phenomenologically (i.e., an objective and specific medical disorder or a recognized pharmacological phenomenon) • Rechallenge satisfactory, if necessary
Probable/likely	• Event or laboratory test abnormality, with reasonable time relationship to drug intake • Unlikely to be attributed to disease or other drugs • Response to withdrawal clinically reasonable • Rechallenge not required
Possible	• Event or laboratory test abnormality, with reasonable time relationship to drug intake • Could also be explained by disease or other drugs • Information on drug withdrawal may be lacking or unclear
Unlikely	• Event or laboratory test abnormality, with time to drug intake • that makes a relationship improbable (but not impossible) • Disease or other drugs provide plausible explanations

#### Serious adverse events that require a notification without delay by the investigator to the sponsor

As per article R.1123-49 of the French Public Health Code (PHC), the investigator must notify the sponsor *without delay on the day when the investigator becomes aware* of any serious adverse event which occurs during a trial as described in Article L.1121-1(1) PHC, except those which are listed in the protocol (see section 10.1.2.2.2) and, if applicable, in the investigator’s brochure as not requiring a notification without delay.

A serious adverse event is any untoward medical occurrence that:

**Table:**

1- Results in death 2- Is life-threatening 3- Requires inpatient hospitalization or prolongation of existing hospitalization 4- Results in persistent or significant disability/incapacity 5- Is a congenital anomaly/birth defect	

Specific features of the protocol

### Other events that require the investigator to notify the sponsor without delay

The expected serious adverse events associated with no specific research procedures or exams. The investigator must notify the sponsor of the following events immediately if they occur within 48 h of treatment.

- Cardiovascular collapse defined as a drop of mean arterial pressure of 25% or more baseline value within 15 min after initiating treatment.
- Cardiac arrest.
- Ventricular tachycardia or ventricular fibrillation.
- Acute myocardial infarction (ST+).
- Severe hypoxemia will be defined as PaO_2_/FiO_2_ < 100 mmHhg.
- Hypotension will be defined as a drop of mean arterial pressure of 25% or more from baseline value within 15 min after introducing the treatment.
- A major bleeding episode that will be defined as:
  - ◦ Epistaxis if it lasts for more than 5 min, if it is repetitive (i.e., two or more episodes of true bleeding, i.e., no spots on a handkerchief, within 24 h), or if it leads to an intervention (packing, electrocautery, etc.).
  - ◦ Gingival bleeding if it occurs spontaneously (i.e., unrelated to tooth brushing or eating) or if it lasts for more than 5 min.
  - ◦ Hematuria if it is macroscopic and if either spontaneous or lasts for more than 24 h after instrumentation (e.g., catheter placement or surgery) of the urogenital tract.
  - ◦ Macroscopic gastrointestinal hemorrhage: at least 1 episode of melena or hematemesis, if clinically apparent.
  - ◦ Rectal blood loss, if more than a few spots.
  - ◦ Hemoptysis, if more than a few speckles in the sputum, or intramuscular hematoma.
  - ◦ Subcutaneous hematoma if the size is larger than 25 cm^2^ or larger than 100 cm^2^ if provoked
  - ◦ Multiple sources of bleeding events

Monitoring of such events is part of the standard of care in ICU patients (e.g., arterial pressure monitoring, hemoglobin level monitoring, blood gas monitoring, etc.).

### Serious adverse events that do not require the investigator to notify the sponsor immediately

These serious adverse events are only recorded in the “adverse event” section of the case report form (e-CRF)

### Adverse events that are “not serious” but which are significant for the safety of participants

Expected non-serious adverse events are:

- Transient and rapidly reversed arterial hypotension
- Mild hypoxemia
- Not severe bleeding

## Abbreviations

SOFA: Sequential organ failure assessment
PaO_2_: Partial arterial oxygen pressure
FiO_2_: Inspired fraction of oxygen
eCRF: Electronic case report form
CRB: Center de Ressources Biologiques
DSMC: Data and Safety Monitoring Committee
APHP: Assistance Publique Hôpitaux de Paris
DRCD: Clinical Research and Development Department
SAE: Serious adverse events
IRB: Institutional review board
