# Supplementary material for: The I-MICRO trial, Ilomedin for treatment of septic shock with persistent microperfusion defects: a double-blind, randomized controlled trial—study protocol for a randomized controlled trial
Source: Trials. 2020 Jul 1;21:601. doi: 10.1186/s13063-020-04549-y (PMC7329442; doi:10.1186/s13063-020-04549-y)
Supplement: Supplementary file 4 — Additional file 4:. Clinical trial authorization for medicinal products for human use [file 13063_2020_4549_MOESM4_ESM.docx]

CLINICAL TRIAL AUTHORIZATION FOR MEDICINAL PRODUCTS FOR HUMAN USE

#
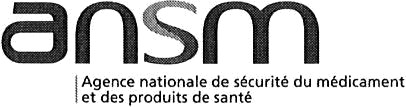
Date: 1


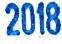

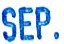


| Clinical Trial Identifiers | | | |
| --- | --- | --- | --- |
| Title | Ilomedine in the Treatment of Septic Shock with Persistent Microperfusion Disorders Double-blind, Randomized, Controlled Multicenter Study | | |
| Promoter | ASSISTANCE PUBLIQUE - HOPITAUX DE PARIS (AP-HP) | | |
| Ref. to be recalled | MEDAECNAT-2018-07-00015 | N° EudraCT | 2018-001709-10 |

| Sender |
| --- |
| ANSM / Product Management Medicines in cardiology, rheumatology, stomatology, endocrinology, gynaecology, urology, pneumology, ENT, allergology / VASC Team |
| File followed by: Kamel Sedkaoui  Phone: 33 (0) 1.55.87.40.84 / Fax 33 (0) 1.55.87.30.53  Mail: kamel.sedkaoui@ansm sante fr |

| Recipient (applicant . name / company / tel.) | |
| --- | --- |
| Elodie LEMADRE  ASSISTANCE PUBLIQUE - HOPITAUX DE PARIS (AP-HP)  01-44-84-17-34 | |
| Mail | [elodie.lemadre@aphp.fr](mailto:elodie.lemadre@aphp.fr) |

| CPP recipient | 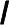 | Mail | 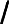 |
| --- | --- | --- | --- |

Having regard to the Public Health Code and in particular Article L. 1123-8, and the regulatory provisions adopted for its application, and having regard to the clinical trial authorisation application file sent to the National Agency for the Safety of Medicines and Health Products (ANSM) ;

Considering the supplements paid by the sponsor dated 07/09/2018 and in particular the protocol of the trial mentioned in the modified object (version 1.1 dated 07/09/2018), following the request of the ANSM ;

The authorisation mentioned in article L. 1123-8 of the Public Health Code is granted for the clinical trial mentioned in the subject.

Cardiology, Rheumatology, Stomatology, Gynaecology, Urology, Pneumology, ENT, Allergy Drugs Directorate

Cardiovascular, Thrombosis, Metabolism, Rheumatology, Stomatology" cluster.

Badis-Lakhdar BENSAAD

Product Manager

I ask you to send any request for modifications concerning this file by email to the following address: ams-

essaysclinicquestQansm.sante.fr . When sending these dossiers, I ask you to make sure that the subject of the message states: AMM/EURDRACT No. for MS submitted for authorisation or for mixed dossiers (including modifications submitted for authorisation and others for information).

### Confidentiality Conłidenti ality

Cetle braIMsm issioJï est ú 1 'allcJal ion cxc lusive du{ des ) destr nalaires c i-dessus meIn ionnêts) ei Th i.s ma nsJn i ssioi is i ntended to the nddressee{s) 1 is1ed above on ly and iøa› cor› t ain peul cont cJ4 ir des inforuaat tons pt ivi légi ées eL'ou corifidei Jtie1 les Si vous iI’ éi es pas le ¡are ferei tial orland coøfideiit i a1 i n fort laation I f › ou are not ltte i merided rec i }a rent , )'ou dcsIiualaire 'oulu ou uhe persoi me iøaudat će pour lui rent ettre ceiie H ansin issioi , vo‹is avez are hereby uo ii fied that you leave reccived ltte doct uren1 by i›i tspake and an) It se, rcçii ce ‹1ociJme Jet par erreur ei tow re ui i lisaiiori, révć1 at ioit, copie on cona i Ja ‹ni ical ion õe son di sc1 osure, copy i rig or couiJnii Jaicat ioJi of the con1enI of llï is łransni ission is p rem thi1ed comenu est i nterdiie . si vous avez reçu ceiie iransJn i ssioii par ct i eu r, veuń1 ez i Jous en If ›'ou have receit ed t lus mausmission by inislake, please call us › niriædiale1)' a nõ i n forJrcr par ić1 ćpilot Je iIninéd rat eineJit et i ous reio‹irner le message oripina1 pa r co‹irrier. rel tirii due origiit al i Jaessa ge hj mat I TlJunk you .

### Mcrci. co6e AEG_FOR004 v03

143/147, bd Anatole France - F-93285 Saint-Denis cedex - UI +33 (0)1 55 87 30 00 - [www.ansm.sante.fr](http://www.ansm.sante.fr/) Page 1 sur 1
