# Supplementary material for: The I-MICRO trial, Ilomedin for treatment of septic shock with persistent microperfusion defects: a double-blind, randomized controlled trial—study protocol for a randomized controlled trial
Source: Trials. 2020 Jul 1;21:601. doi: 10.1186/s13063-020-04549-y (PMC7329442; doi:10.1186/s13063-020-04549-y)
Supplement: Supplementary file 5 — Additional file 5:. Protection of persons committee. [file 13063_2020_4549_MOESM5_ESM.docx]

#### President:

PROTECTION OF PERSONS COMMITTEE

South East V

Grenoble le, 30/10/2018

**AP-HP - Hôpital Saint-Louis**

### Dr Daniel ANGLADE

#### Vice-President:

**A-M. BENOIT-BALLANSAT**

#### Secretary General:

**Dr Arnaud SEIGNEURIN**

#### Deputy Secretary General:

**Dr Dominique CHARLETY**

#### Treasurer:

**62. Réf. CPP: 18-APHP-06** Ref Etude : I-MICRO - P170924J N° IDRCB: 2018-001709-10

Document provided

## DRCI

Madame LEMADRE ELODIE

*Project manager of the promotion department*

1, avenue Claude Vellefaux **75010 PARIS**

**Mme S. CALVINO-GUNTHER**

#### Site internet

[http://www.cppsudest5.fr](http://www.cppsudest5.fr/)

Secrétariat :

[***cppsudest5@chu-grenoble.fr***](mailto:cppsudest5@chu-grenoble.fr)

**F. LUCZAK**

**N. CESTARO**

#### Tel : 04 76 76 57 83

Fax : 04 76 76 51 77

GSM : 06 50 85 79 26

Domiciliation:

🖃 **CHU de Grenoble Comité de Protection des Personnes**

**Mailing address*:***

**CS 10217**

**38043 GRENOBLE Cedex 9**

Geographic address:

R.C. Haut Hall Vercors

Bd de la Chantourne 38700 LA TRONCHE

N° SIRET : 130 016 017 00019 N°APE : 8412Z

Protocol Version N° and date : n° 1.0 du 26/06/2018 Patient information letter N° 1.0 du 12/04/2018 Consent form N° 1.0 du 12/04/2018

Protocol_V1_2_CPP_et_ANSM 12_09_2018 EL_FD_ZTB charterCSI_v1.1_du_24_09_2018_sign_prar_Pr_Luyt courrier_CPP_12_09_2018_sign_ list_investigators_24092018 answers_to_CPP_V1_0_du_24_09_2018_EL_ML_ZTB Additional_Document_CPP_V1.1_20180912_ZTB_EL justif_adequation_means_MED_20180924_EL

nifc_RI_major_out_state_express_closest_consent_V1.0_20180912_EL_ZTB_ 20180317

nifc_RI_major_V1.1_20180912_EL nifc_RI_pursuit_V1_0_20180912_EL_17092018_ZTB

resum V1.2_12_09_2018_English_modif_CPP_EL_ZTB CTA_PDF_V1.1_du_24_09_2018_sign_

#### Sponsor: AP-HP- Hôpital Saint- 75010 PARIS

#### Principal Investigator: Dr François Dépret - Paris – Biomedical Research Type: TYPE 1 MEDICAMENT

#### Objet: **Favourable opinion**

#### **Madam,**

**The Chairman of the Committee,** gave a **FAVOURABLE** opinion, dated 18 October 2018, for the protocol, received on 25/07/2018.

***« Ilomedin for treatment of septic shock with persistent microperfusion defects », a double-blind, randomized controlled trial: I-MICRO trial***

after consultation with the rapporteurs and consideration by the Committee at its meetings on 30/08/2018 and 18/10/2018.

The promoter is Assistance Publique - Hôpitaux de Paris (AP-HP) and by delegation: The promoter is Assistance Publique - Hôpitaux de Paris (AP-HP) and by delegation: DRCI - Hôpital Saint-Louis - 1, avenue Claude Vellefaux - 75010 PARIS

The principal investigator is Dr François Dépret - 10 rue Bouchardon - 75010 PARIS - France.

RPPS: 10100815561

Associate Investigators are listed.

The persons who deliberated on the project are:

| 1st college:   1. **– Biomedical research**   *Holder:*  Mme PARIS Adeline  *Substitute:*  Dr DAVID-TCHOUDA Sandra  Mme Dole Marjorie   1. **– General practitioner:**   *Nothin:*   1. **– Hospital pharmacist:**   *Holder*: Mme CHARLETY Dominique   1. **– Nurse:**   *Nothin* | 2nd college:   1. **- Persons qualified as "ethical"** *Holder:* M. BASSET Pierre *Substitute*: Mme LOPEZ Mélaine 2. **- Psychologist**   *Holder:* Mme NAEGELE Bernadette  *Substitute*: M. BOUATI Noureddine   1. **– Social worker**   *Nothin:*   1. **- Personnes qualifiées "juridique"**   *Holder:* Mme BENOIT-BALLANSAT Anne-Marie Mme DALL’AGLIO BRANMBILLA Géraldine *Substitute*: Mme ANGLADE Prune  Mme BARTHE-BOUGENAUX Dominique   1. **– Représentants d'association agréée de malades et d'usagers du système de santé**   *Holder:* Mme AUZIMOUR Renée |
| --- | --- |

*No deliberative member of the Committee is affected by a conflict of interest.*

Please believe, Madam, in the expression of my distinguished greetings.


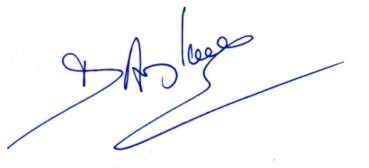


**The President**

**Dr Daniel ANGLADE**
